# Comparison of the Effectiveness of Transepidemal and Intradermal Immunization of Mice with the Vacinia Virus

**DOI:** 10.32607/actanaturae.11857

**Published:** 2022

**Authors:** S. N. Shchelkunov, A. A. Sergeev, K. A. Titova, S. A. Pyankov, E.V. Starostina, M. B. Borgoyakova, L. A. Kisakova, D. N. Kisakov, L. I. Karpenko, S. N. Yakubitskiy

**Affiliations:** State Research Center of Virology and Biotechnology VECTOR, Rospotrebnadzor, Koltsovo, Novosibirsk region, 630559 Russia

**Keywords:** orthopoxviruses, vaccinia virus, skin scarification, intradermal injection, antibodies, T cells

## Abstract

The spread of the monkeypox virus infection among humans in many countries outside of Africa, which started in 2022, is now drawing the attention of the medical and scientific communities to the fact that immunization against this infection is sorely needed. According to current guidelines, immunization of people with the first-generation smallpox vaccine based on the vaccinia virus (VACV) LIVP strain, which is licensed in Russia, should be performed via transepidermal inoculation (skin scarification, s.s.). However, the long past experience of using this vaccination technique suggests that it does not ensure virus inoculation into patients’ skin with enough reliability. The procedure of intradermal (i.d.) injection of a vaccine can be an alternative to s.s. inoculation. The effectiveness of i.d. vaccination can depend on the virus injection site on the body. Therefore, the aim of this study was to compare the development of the humoral and cellular immune responses in BALB/c mice immunized with the LIVP VACV strain, which was administered either by s.s. inoculation or i.d. injection into the same tail region of the animal. A virus dose of 105 pfu was used in both cases. ELISA of serum samples revealed no significant difference in the dynamics and level of production of VACV-specific IgM and IgG after i.d. or s.s. vaccination. A ELISpot analysis of splenocytes from the vaccinated mice showed that i.d. administration of VACV LIVP to mice induces a significantly greater T-cell immune response compared to s.s. inoculation. In order to assess the protective potency, on day 45 post immunization, mice were intranasally infected with lethal doses of either the cowpox virus (CPXV) or the ectromelia virus (ECTV), which is evolutionarily distant from the VACV and CPXV. Both vaccination techniques ensured complete protection of mice against infection with the CPXV. However, when mice were infected with a highly virulent strain of ECTV, 50% survived in the i.d. immunized group, whereas only 17% survived in the s.s. immunized group. It appears, therefore, that i.d. injection of the VACV can elicit a more potent protective immunity against orthopoxviruses compared to the conventional s.s. technique.

## INTRODUCTION

During mass vaccination, virus preparations are administered either intramuscularly or subcutaneously, since these techniques are the simplest to perform, ensure accurate vaccine dosage, and do not require a highly qualified staff. However, these body tissues where a vaccine is delivered are immune-poor and usually do not elicit a long-lasting, potent immune response to the administered vaccine [1 , 2, 3]. Nonetheless, next-generation smallpox vaccines (including the best studied MVA strain) continue to be typically administered intramuscularly or subcutaneously [4, 5].

Skin immunization is a promising alternative to the conventional subcutaneous and intramuscular administration paths. The reason is that not only does the skin act as a physical barrier, preventing penetration of infectious agents into the body, but it also has evolved to become a highly active immune organ. The skin contains various types of dendritic cells, and these professional antigen-presenting cells (APCs) can recognize, assimilate, and process antigens. Importantly, these dendritic cells underpin the necessary association between the innate and adaptive immune responses by migrating into the skin, draining lymph nodes and presenting antigens to T and B cells, thus inducing a pathogen-specific protective immunity. Furthermore, these highly specialized APCs possess significant plasticity, which is modulated by immune signals emanating from other virus-infected skin cells (including keratinocytes, fibroblasts, melanocytes, mast cells, etc.) [1, 2, 3, 6].

Transepidermal immunization is historically the first-ever vaccination technique and originates from variolation (variola inoculation). The procedure involved placing infectious material from smallpox patients into skin incisions (skin scarification, s.s.) made in healthy patients. In the late 18th century, E. Jenner proposed inoculating the contents of pustules from people infected with the cowpox rather than infectious material from smallpox patients. This procedure became known as vaccination (vaccine inoculation). Transepidermal immunization was performed using a scalpel, a lancet, or specialized bifurcated needles. Although this vaccination method has made it possible to eradicate smallpox, reliability in delivering viral material into the skin was never sufficiently high [1]. Furthermore, this procedure can be accompanied by the growth of bacterial microflora in the damaged skin [7].

In 1909, C. Mantoux [8] proposed to make intradermal injections using a syringe with a standard needle. This method became actively used in the administration of the BCG anti-tuberculosis vaccine, which was developed in 1921. A century later, the conventional Mantoux technique for intradermal injection is now used only to administer a small number of vaccines. The reason is that this injection method is not easy to perform: the antigen can either be delivered too deep under the skin, or the vaccine may leak out of the injection site [9]. Therefore, staff needs to be specially trained and have experience making such injections.

The recently conducted animal experiments and clinical trials on volunteers have consistently shown that intradermal vaccination elicits a more potent immune response compared to the conventional intramuscular or subcutaneous varieties [10, 11, 12]. Furthermore, intradermal vaccination can ensure a robust immune response at a lower vaccine dose [1, 12], which is also important in the case of mass vaccination, when a large number of vaccine doses need to be produced.

Individual studies report the results of experiments on laboratory animals comparing the effectiveness of the immune response against the vaccinia virus (VACV) delivered by different methods: intramuscularly, subcutaneously, intradermally, intraperitoneally, etc. Intradermal injection of VACV has consistently ensured a more robust antiviral immune response compared to other vaccination techniques [10, 13]. The results in these studies also depended on the analyzed VACV strains and virus doses used.

Liu L. et al. [14] demonstrated that inoculating the VACV WR strain highly pathogenic for mice into the scarified tail skin of mice can elicit an immune response stronger than that observed after intradermal injection of this virus into the low back of mice. Skin thickness is known to vary depending on the region of the body [2]. Therefore, the effectiveness of intradermal vaccination can hinge on the virus injection site. All these facts indicate that comparative studies are needed in order to determine how the technique used for inoculating the VACV strain into the skin within the same body area affects the immune response dynamics and level.

The VACV LIVP strain used to design the first-generation smallpox vaccine in Russia [15] was the study object. The study aimed to compare the humoral and T cell-mediated immune responses to vaccination of BALB/c mice with the VACV LIVP strain inoculated into the same tail region by scarification (transepidermally) or by injection with a needle and a syringe using the Mantoux technique (intradermally).

## EXPERIMENTAL

**Viruses and cells**

The clonal variant 14 of the VACV LIVP strain [16], cowpox virus (CPXV) strain GRI-90 [17], and ectromelia virus (ECTV) strain K-1 from the Virus collection and African green monkey kidney cell culture CV-1 from the Cell culture collection of the SRC VB VECTOR were used in this study. The viruses were grown and titrated in the CV-1 cell culture using the procedures described previously [15].

**Animals**

Female BALB/c mice aged 6–7 weeks (weight, 16–19 g) procured from the husbandry of the SRC VB VECTOR were used for the experiments. The experimental animals were fed a standard diet with ad libitum access to water, in compliance with the veterinary regulations and the guidelines for humane handling and use of animals in research. Animal manipulations were approved by the Bioethics Committee of the SRC VB VECTOR (Protocol No. 01-04.2021 dated April 22, 2021).

**Infection of mice**

The animals were immunized by intradermal injection (i.d.) or skin scarification (s.s.) using the VACV LIVP at a dose of 105 plaque forming units (pfu).

For the i.d. injection, the injection site (the dorsal side of tail, ~ 1 cm from the tail base) was pre-disinfected with 70% ethanol; a needle 30G (0.3 × 13 mm) connected to a syringe was inserted at a small angle, with the needle bevel facing up, to a depth of ~ 2–3 mm under the superficial level of the epidermis. Viral material or saline (control group), 20 μl, was injected slowly, with the expectation that the top skin layers will get delaminated due to the pressure of the fluid (blanching of the skin spreading to both sides of the injection site was indication that the fluid had got into the intradermal space). After the injection, the needle was withdrawn slowly and the injection site was disinfected with 70% ethanol.

For immunization using the s.s. technique, the inoculation site (the dorsal side of the tail, ~ 1 cm from the tail base) was pre-disinfected with 70% ethanol. Once the ethanol had evaporated, 10 skin incisions were made using a needle 26G (0.45 × 16 mm) within the superficial layer of the epidermis. Viral material or saline (5 μl) was immediately placed onto the damaged skin area and was let to be adsorbed by the skin.

Each group consisted of 36 mice.

**Sampling of biomaterials from the experimental animals**

After the immunization (7, 14, 21, and 28 days post immunization (dpi)) with the VACV, blood samples were collected from the retro-orbital venous sinus of mice (six animals from each group) by puncturing the sinus with a needle 23G (0.6 × 30 mm); the animals were then euthanized by cervical dislocation. Spleens for splenocyte isolation were removed under sterile conditions using forceps and surgical scissors and placed into the transport medium.

Serum specimens were obtained from the individual blood samples of mice by centrifugation of blood cells. Mouse serum specimens were stored at –20°C.

On 42 dpi with VACV, blood samples were collected from the retro-orbital venous sinus intravitally in mice (12 animals from each group) and individual serum specimens were obtained using the procedure described above.

**Assessment of the protective potency in immunized mice**

On 45 dpi, the groups of virus-immunized and control animals were intranasally (i.n.) infected with CPXV GRI-90 at a dose of 300 LD_50_ (3.2 × 10^6^ pfu) (six animals per group) or with ECTV K-1 at a dose of 300 LD_50_ (7.3 × 10^3^ pfu) (six animals per group). The animals were followed for clinical signs of infection and mortality for 14 days.

The mice were individually weighed every two days. The arithmetic mean body weight of the mice in each group at every time point was calculated and expressed as a percentage of the initial weight. Data were obtained for the group of animals immunized with VACV LIVP, as well as the non-immunized and not-infected group of mice (negative control) and those infected with CPXV GRI-90 or ECTV K-1 (positive control).

**Splenocyte isolation**

The spleens collected from the immunized mice were mashed onto 70-μm and 40-μm cell strainers (BD Falcon™, Tewksbury, MA, USA). Splenocytes were treated with a red blood cell lysis buffer (ACK Lysis Buffer, Sigma, St. Louis, MO, USA); then, the cells were washed with a completed RPMI 1640 medium and suspended in the completed RPMI 1640 medium with 10% fetal bovine serum, 2 mM L-Gln, and 50 μg/mL gentamycin. The cells were counted with a TC20™ automated cell counter (Bio-Rad, Hercules, CA, USA).

**IFN-γ ELISpot assay**

The assays were performed using the mouse IFN-γ ELISpot kit (R&D Systems, Inc., Minneapolis, MN, USA) according to the manufacturer’s instructions. The splenocytes were plated (100 μL/well) in duplicates 5 × 10^6^ cells/mL and stimulated by a mixture of peptides (corresponding to VACV-specific BALB/c mice H2-d restricted epitopes): SPYAAGYDL, SPGAAGYDL, VGPSNSPTF, KYGRLFNEI, GFIRSLQTI, and KYMWCYSQV [18]. The pooled peptides (100 μL/well) were added at a concentration of 20 μg/mL for each peptide. Non-stimulated and concanavalin A (Con A, 5 μg/mL) stimulated splenocytes were used as the negative and non-specific positive controls, respectively. After an 18-h stimulation period at 37°C in 5% CO_2_, the cells were discarded and the plates were incubated for 2 h at 37°C in the presence of anti-IFN-γ detection antibodies.

The plates were washed and the spots were revealed by adding the streptavidin-conjugated alkaline phosphatase and the BCIP/NBT (5-bromo-4-chloro- 3′-indolylphosphate/nitro-blue tetrazolium) substrate. The reaction was stopped by washing the plates with distilled water. The number of IFN-γ-producing cells was counted using an ELISpot reader (Carl Zeiss, Jena, Germany).

**Enzyme-linked immunosorbent assay of the serum samples**

ELISA of individual mouse serum specimens was performed according to the procedure described earlier [15]. The purified VACV LIVP preparation was used as an antigen. The geometric means of the logarithms of the reciprocal titers of VACV-specific IgM and IgG in the study groups were determined, and the confidence intervals for a 95% confidence level were calculated.

**Statistics**

The data were analyzed with the GraphPad Prism 9.0 software (GraphPad Software, Inc., San Diego, CA, USA). The results are expressed as a geometric mean with GSD. Data throughout the study were analyzed using repeated-measures two-way ANOVA with the Geisser-Greenhouse correction. Multiple comparisons were performed using a Tukey test. The statistical analysis was conducted at a 95% confidence level. A P value less than 0.05 was considered statistically significant.

## RESULTS

**Intradermal injection of VACV LIVP to mice induces a stronger cell-mediated immune response compared to virus inoculation by skin scarification**

Changes in the T-cell immune response in LIVP-vaccinated BALB/c mice over time were investigated using the IFN-γ ELISpot technique. The mice were split into several groups (six animals per group). The animals were inoculated with the VACV LIVP either i.d. (1 cm from the tail base) or s.s. (1 cm from the tail base) at a dose of 105 pfu/animal. The spleens for performing ELISpot assay were removed individually from six animals in each study group on 7, 14, 21, and 28 dpi. Intact (non-immunized) mice were used as control.

**Fig. 1:**
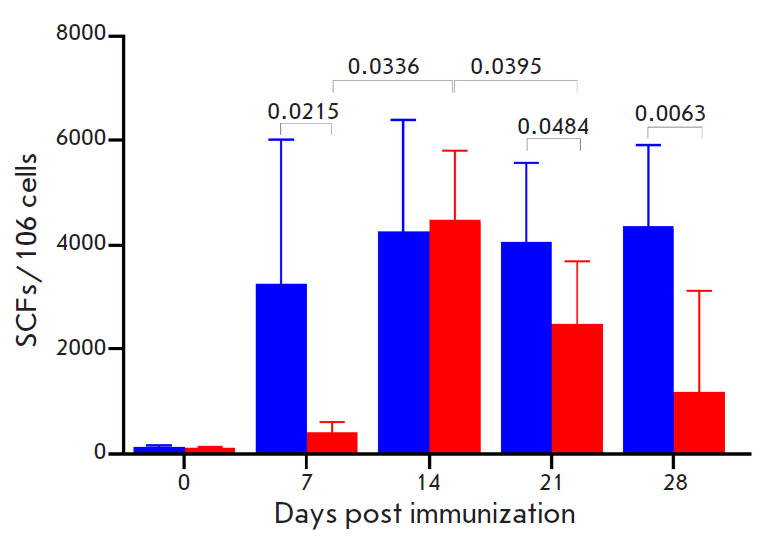
Assessment of T cell-mediated immunity in BALB/c mice immunized with VACV LIVP (six mice per group) by IFN-γ ELISpot assay. Splenocytes were stimulated with a pool of virus-specific peptides during 24 h. Blue bars – i.d. injection of the VACV LIVP; red bars – s.s. inoculation of the VACV LIVP. The diagrams show the geometric mean with GSD. The Y axis shows the number of spots (the number of IFNγ-producing cells) per 106 splenocytes. Day 0 – the level of T cell-mediated immune response for non-immunized mice. The statistical analysis was performed using the GraphPad Prism 9.0 software. *P *values are above horizontal brackets

The intensity of the T cell-mediated immune response in the immunized mice was determined according to the number of splenocytes producing IFN-γ in response to the stimulation with peptides from the immunodominant VACV proteins [19]. The results shown in Fig. 1 demonstrate that a potent VACV-specific T cell-mediated immune response was elicited in all immunized mice. Meanwhile, the splenocytes in the control animals did not produce IFN-γ.

After s.s. inoculation of VACV LIVP, on 7 dpi only a low level of VACV-specific T cell-mediated immunity was induced in mice, reaching its maximum on 14 dpi and declining significantly on 21 and 28 dpi (Fig. 1).

After i.d. injection, an intensive T cell-mediated immune response developed in mice as early as on 7 dpi, slightly increased by 14 dpi, and remained high during the entire follow-up period (up to 28 dpi).

On days 7, 21, and 28, the level of T cell response in i.d. vaccinated mice significantly exceeded that in the groups of mice s.s. inoculated with VACV LIVP (Fig. 1).

**No difference in the dynamics of developing humoral immunity in mice in response to inoculation of VACV LIVP by intradermal injection or skin scarification was revealed**

Individual blood samples were collected from the retro-orbital venous sinus in mice on 7, 14, 21, 28, and 42 dpi to obtain serum specimens, which were then analyzed by ELISA; the preparation of VACV LIVP virions was used as an antigen.

**Fig. 2:**
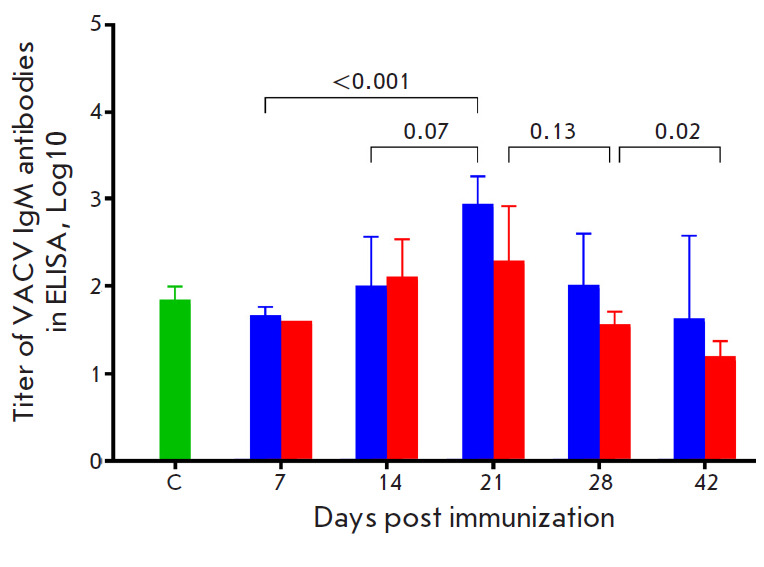
Concentration of VACV-specific IgM in the serum samples of mice immunized with VACV LIVP at a dose of 105 pfu determined by ELISA. Blue bars – i.d. injection of the VACV LIVP; red bars – s.s. inoculation of the VACV LIVP. C (control) – serum samples from mice that received saline. The diagrams show the geometric mean with GSD. The statistical analysis was performed using the GraphPad Prism 9.0 software. *P *values are above horizontal brackets

**Fig. 3:**
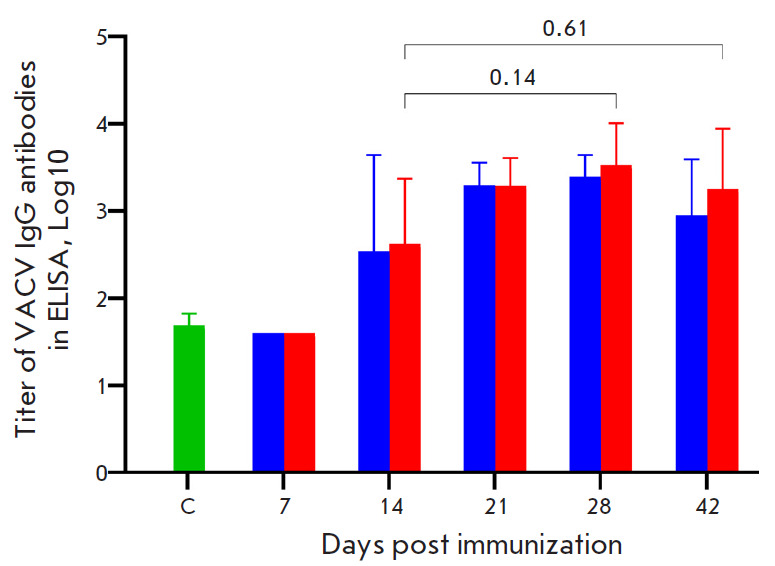
Concentration of VACV-specific IgG in the serum samples of mice immunized with the VACV LIVP at a dose of 105 pfu determined by ELISA. Blue bars – i.d. injection of the VACV LIVP; red bars – s.s. inoculation of the VACV LIVP. C (control) – serum samples from mice that received saline. The diagrams show the geometric mean with GSD. The statistical analysis was performed using the GraphPad Prism 9.0 software. *P *values are above horizontal brackets

Serum samples from six animals were analyzed at each time point in each group. The geometric means of the logarithms of reciprocal titers of VACV-specific IgM and IgG were calculated. The maximum level of VACV-specific IgM was observed in mice on 21 dpi (Fig. 2), while the maximum level of VACV-specific IgG production was observed on 28 dpi (Fig. 3).

**Intradermal injection of VACV LIVP to mice provides greater protective potency than inoculation of this virus by skin scarification**

In order to understand how the levels of humoral and cell-mediated immunity developing in response to the immunization of the mice with the VACV LIVP affect their protective potency against a challenge with a lethal orthopoxvirus infection, the mice were i.n. infected with lethal doses of CPXV GRI-90 (six animals per group) or ECTV K-1 (six animals per group) on day 45 post i.d. or s.s. inoculation of the VACV LIVP. The mice were followed up for 14 days; clinical manifestations of the infection and death of the animals were documented. Every two days, mice were weighed to determine the dynamics of body weight change.

**Fig. 4:**
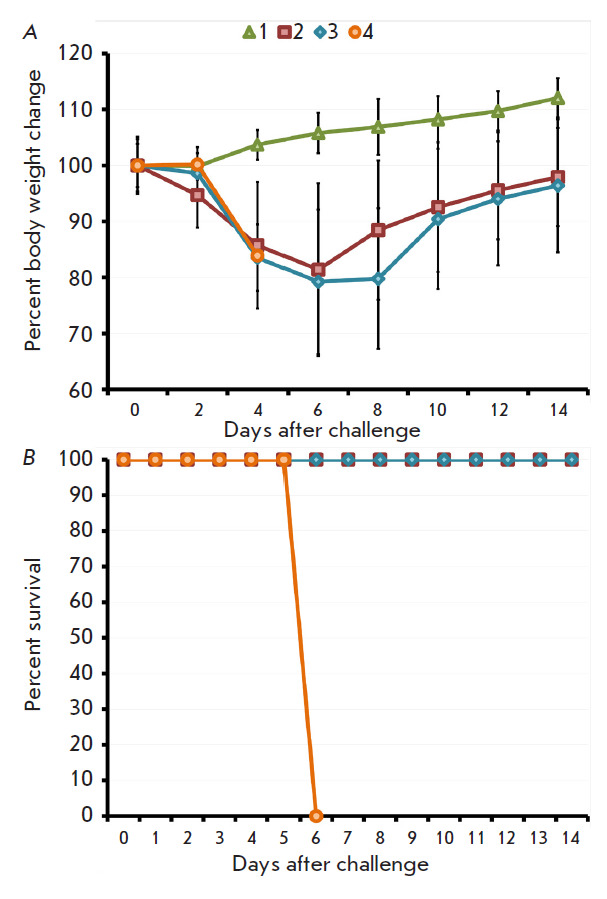
The dynamics of body weight change (*A*) and death of mice (*B*) immunized with VACV LIVP at a dose of 105 pfu after i.n. infection with CPXV GRI-90 at a dose of 300 LD_50_. Data for groups consisting of six animals immunized using the s.s. (2) or i.d. (3) technique, as well as the groups consisting of non-immunized animals either non-infected (1) or infected with CPXV GRI-90 (4), are shown

After the mice had been infected i.n. with CPXV at a dose of 3.2 × 10^6^ pfu (300 LD_50_), the animals in the study groups started displaying signs of disease and their body weight declined transiently on days 4–8 without statistically significant differences (Fig. 4A). All the animals in the positive control group had died by day 6, while all the mice in study groups had recovered (Fig. 4B).

**Fig. 5:**
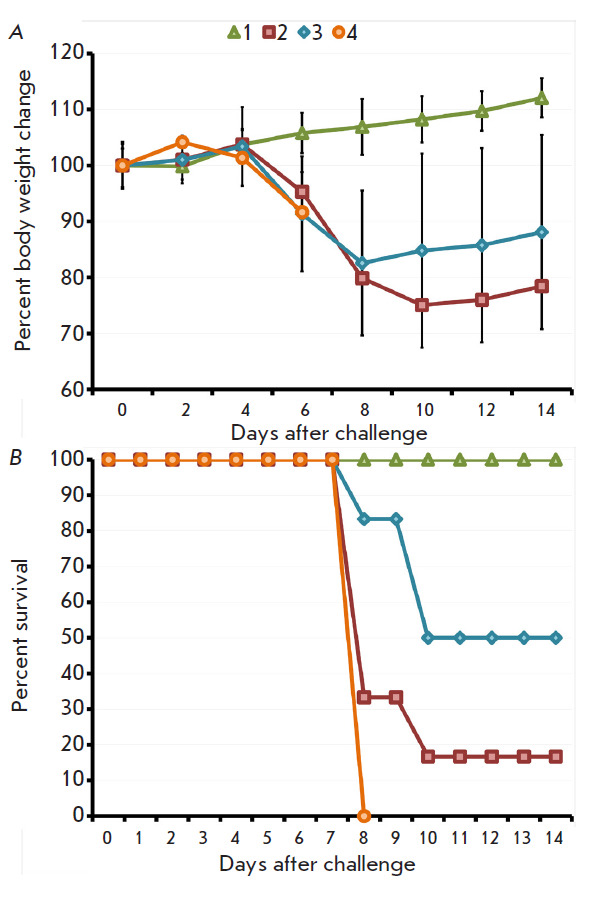
The dynamics of body weight change (*A*) and death of mice (*B*) immunized with VACV LIVP at a dose of 105 pfu after i.n. infection with ECTV K-1 at a dose of 300 LD_50_. Data for groups consisting of six animals immunized using the s.s. (2) or i.d. (3) technique, as well as the groups consisting of non-immunized animals either non-infected (1) or infected with ECTV K-1 (4), are shown

After the mice had been infected i.n. with the highly pathogenic ECTV at a dose of 7.3 × 10^3^ pfu (300 LD_50_), signs of disease were observed in study groups on days 6–10 and the animals’ body weights declined transiently without statistically significant differences (Fig. 5A). All the animals in the positive control groups had died by day 8. Half of the mice in the group of animals vaccinated by i.d. injection survived, while only 17% of the animals vaccinated by s.s. inoculation of the virus survived (Fig. 5B).

## DISCUSSION

The skin possesses properties that make it an excellent site for vaccination. It is an immune-rich organ and contains components that efficiently induce both humoral and cell-mediated immunity in response to infection/vaccination [1 , 2, 3]. There are two techniques for cutaneous vaccination: the historically older method of transepidermal inoculation or skin scarification (s.s.) and the technique of intradermal injection (i.d.), which was proposed in the early 20th century [8]. Each of these methods has advantages and shortcomings.

The technique of s.s. inoculation is relatively simple, but the skin cover is disrupted when used in that way. Thus, a local inflammatory response is induced and it is difficult to ensure dosage accuracy. The i.d. injection using a needle and a syringe causes minimal skin damage and allows one to dose the vaccine and inject it into the target skin layer more accurately.

Despite the long history of using both the s.s. and i.d. vaccination techniques, no fully correct comparison of the immunogenic and protective effectiveness of these two methods upon inoculation of the VACV in animal models has been performed. Such a conclusion can be drawn because in most studies comparing the s.s. and i.d. techniques, the VACV was inoculated into different body sites of laboratory mice [19]. The results of our preliminary experiments have shown that the body site of mice into which the virus preparation is inoculated significantly affects the immune response level upon i.d. injection of the VACV. In order to eliminate this effect, we have compared the s.s. and i.d. techniques when the same dose of the VACV is inoculated into the same site at the mouse tail.

BALB/c mice and the VACV LIVP strain were used as study objects. The VACV LIVP at a dose of 105 pfu was inoculated either i.d. or s.s. to mice into the tail skin (1 cm from the tail base). For each of the two studied vaccination methods, blood was sampled from the retro-orbital venous sinus in six animals at each time point (7, 14, 21, and 28 dpi) and individual serum samples for the analysis of the levels of VACV-specific antibodies were obtained. Next, spleens were removed from each animal to isolate splenocytes and perform a IFN-γ ELISpot assay. Intact (non-immunized) mice were used as control.

The intensity of the T cell-mediated immune response in immunized mice was determined according to the number of splenocytes producing IFN-γ in response to stimulation with peptides from the immunodominant VACV proteins (Fig. 1). Only a low level of VACV-specific T cell-mediated response was induced by s.s. inoculation of the VACV LIVP on 7 dpi; it reached its maximum on 14 dpi and began declining significantly by 21 and 28 dpi. After i.d. injection, an intensive T cell-mediated immune response developed in mice as early as on 7 dpi and remained so during the entire follow-up period (up to 28 dpi). On 7, 21, and 28 dpi, the level of the T cell response in i.d.-vaccinated mice significantly exceeded that in the groups of mice s.s. inoculated with VACV LIVP (Fig. 1). Hence, i.d. immunization with the VACV LIVP induces a more potent and lansting T cell-mediated immune response in mice compared to s.s. vaccination.

In the remaining mice in the study and control groups (12 animals per group), blood was sampled intravitally from the retro-orbital venous sinus on 42 dpi, and individual serum samples were obtained. ELISA of all the serum samples of the immunized mice revealed no statistically significant difference in the dynamics and level of production of VACV-specific IgM (Fig. 2) and IgG (Fig. 3) after both the i.d. and s.s. vaccinations. The maximum IgM and IgG levels were observed on 21 and 28 dpi, respectively.

In order to assess the protective immunity that developed as a result of i.d. or s.s. vaccination, six mice per group were infected i.n. with highly lethal doses of CPXV GRI-90 or ECTV K-1 on 45 dpi. Both vaccination methods were found to completely protect mice against infection with CPXV at a dose of 300 LD_50_ (Fig. 4). However, the vaccinated animals had only partial protection after being i.n. infected with a highly virulent ECTV (300 LD_50_), which is relatively evolutionarily distant from the VACV and CPXV [20] (Fig. 5). Meanwhile, 50% of the mice immunized by i.d. injection survived; the percentage of surviving mice immunized by s.s. inoculation was 17%.

These findings allow us to infer that, although humoral immunity makes the greatest contribution to the protection against a challenge with the orthopoxvirus infection [21 , 22, 23], the level of cell-mediated immunity that develops in response to vaccination is also important. A conclusion can also be drawn that intradermal injection of the VACV can ensure a more potent protective immunity compared to the conventional skin scarification technique because of the stronger T cell-mediated response.

The results obtained in this study differ from the findings published earlier by T.S. Kupper et al. [14, 19], who revealed that the VACV exhibits a higher immunogenicity and protectivity upon s.s. immunization of mice compared to the i.d. and other routes of injection of the virus. In those studies, C57BL/6 mice were immunized with the non-replicating VACV MVA strain and protectivity against a lethal respiratory challenge with the VACV WR strain was assessed. For different routes of administration of the viruses, different body parts of mice were challenged.

A different, BALB/c, line of mice was used in our study, and the animals were immunized with the replicating VACV LIVP strain. The protectivity of the immunized mice against a lethal respiratory challenge with the heterologous orthopoxviruses CPXV and ECTV was assessed. Preliminary experiments have revealed that the immunogenicity of the VACV LIVP strain differs significantly upon i.d. injection of the virus into different body sites of mice. Therefore, the VACV LIVP strain was injected into the same region of mouse tail skin in order to properly compare the efficacies of the s.s. and i.d. routes of immunization. This fact seems to be responsible for the discrepancies between our results and the data published previously [14, 19].

The advances in modern techniques of intradermal injection of vaccines will simplify this promising approach to antiviral immunization and increase its reliability [1, 2, 3, 24].

## Abbreviations

CPXV: cowpox virus
ECTV: ectromelia virus
VACV: vaccinia virus
pfu: plaque forming units
i.d.: intradermal
s.s.: skin scarification
dpi: day post immunization
i.n.: intranasal
LD50: 50% lethal dose of virus
